# Efficacy of high-intensity focused ultrasound for treating upper and lower eyelid sagging

**DOI:** 10.1016/j.jpra.2025.10.036

**Published:** 2025-11-03

**Authors:** Yousun Hwang, Jin-Hyun Kim, Han Earl Lee, Isaac Wong Kai Jie, Jong Keun Song, Arash Jalali, Isabella Rosellini, Jesper Thulesen, Massimo Vitale, Eun-Jae Kim, Kyu-Ho Yi

**Affiliations:** a Haedrin Clinic, Center for Aesthetic Medicine, Seoul, Korea; b You and I Clinic, Seoul, Korea; c Opening Plastic Surgery Clinic, Korea; d The Artisan Clinic Private Limited, 435 Orchard Road, #20-03, Singapore 238877; e Pixelab plastic surgery clinic, Seoul, Korea; f One Clinic MD, Vancouver, Canada; g Avery Beauty Clinic and Avena Aesthetics Indonesia; h Capitol Eye Clinic/Clinic Aesthetica, Copehagen, Denmark; i Private practice in Bologna, Italy; j Classys Inc., Seoul, Korea; k Division in Anatomy and Developmental Biology, Department of Oral Biology, Human Identification Research Institute, BK21 PLUS Project, Yonsei University College of Dentistry, 50-1 Yonsei-ro, Seoul, 03722, Republic of Korea

**Keywords:** High-intensity focused ultrasound, Eyelid sagging, Noninvasive rejuvenation, Skin tightening, Periocular treatment

## Abstract

**Introduction:**

Eyelid sagging is a frequent concern among aging patients, yet achieving noticeable improvement with noninvasive methods remains challenging.

**Objective:**

This prospective cohort study evaluated the efficacy and safety of high-intensity focused ultrasound (HIFU) for improving upper and lower eyelid sagging.

**Methods:**

Thirty-four Korean women (aged 31–67 years) with mild to moderate eyelid laxity underwent a single session of HIFU (ULTRAFORMER MPT) using a 2.0-mm, 4-MHz probe, delivering 120 shots to the periocular region. Outcomes were assessed at 12 weeks via a four-point scale rated by blinded clinicians and patients. Eyelid length was measured using standardized photography.

**Results:**

Mean eyelid length decreased by 0.94 ± 0.34 mm (*p* < 0.0001). Clinical improvement was reported by 76 % of patients and recognized by clinicians in 59 % of cases. The mean scores for overall improvement, eyelid tightening, and crow’s feet reduction were 2.25 ± 0.3, 2.10 ± 0.4, and 1.95 ± 0.3, respectively. Mild pain and erythema were the only reported side effects, resolving within 3–4 days.

**Conclusion:**

HIFU is a safe and effective noninvasive option for improving both upper and lower eyelid sagging, with high patient satisfaction and minimal adverse effects.

## Introduction

The periocular area exhibits the earliest and most noticeable signs of facial aging.^1^ The eyelid skin, being the thinnest on the face,^2^ undergoes progressive dermal atrophy characterized by a reduction in elastic fibers, collagen degradation, and an accumulation of elastin-degrading enzymes, ultimately resulting in laxity and sagging.^3^ These changes not only alter facial aesthetics but also have psychosocial implications, leading many patients to seek rejuvenation treatments.^4^

Traditionally, surgical procedures such as blepharoplasty, levator resection, and brow lifting have been used to correct eyelid sagging.^5^ However, due to the invasive nature, extended downtime, and risk of complications, there is increasing demand for minimally or noninvasive alternatives. A range of energy-based devices and injectable techniques have been introduced for skin tightening and rejuvenation, including botulinum toxin, fillers, radiofrequency, and laser treatments.^6^^,^^7^

Among these modalities, high-intensity focused ultrasound (HIFU) has gained widespread attention for its capacity to induce neocollagenesis and skin tightening through thermally induced coagulative zones in the dermis and superficial muscular aponeurotic system (SMAS).^8^^,^^9^ Clinical and histologic studies have demonstrated that HIFU improves skin elasticity and firmness in the face and neck with a favorable safety profile.^10^

Despite these advances, its clinical efficacy and safety for treating the thin and delicate eyelid skin have not been fully validated.^8^^,^^11^ The eyelid area poses distinct anatomical challenges, including a small treatment field, proximity to the globe, and a higher risk of thermal injury.

Therefore, the objective of this prospective cohort study was to evaluate the efficacy and safety of high-intensity focused ultrasound (HIFU) for improving upper and lower eyelid sagging. This study aims to provide quantitative and qualitative evidence supporting HIFU as a noninvasive alternative to surgical blepharoplasty for periocular rejuvenation.

## Materials and methods

### Patients

Thirty-four Korean women aged 31 to 67 years (Fitzpatrick skin type III and IV) with mild to moderate eyelid skin laxity were enrolled in this prospective cohort study. This study was conducted at Haedrin Clinic (Seoul, South Korea) between January and September 2023, in accordance with the principles of the Declaration of Helsinki. All participants provided written informed consent before participation.

Inclusion criteria were: Female patients aged 30–70 years, Fitzpatrick skin types III–IV; presence mild to moderate eyelid skin laxity.

Exclusion criteria were:

(1) Active infection or inflammatory skin disease in the periocular area;

(2) Pregnancy or lactation;

(3) History of keloidal or hypertrophic scarring;

(4) Previous surgical or energy-based aesthetic procedures involving the periocular area within the past 12 months;

(5) Presence of a pacemaker or other implanted electronic devices; and

(6) Inability to provide informed consent.

### Treatment protocol

Topical anesthesia was achieved by applying a 5 % lidocaine–prilocaine cream to the upper and lower eyelids and lateral canthal area for 40 min after gentle facial cleansing.

**Figure 1:**
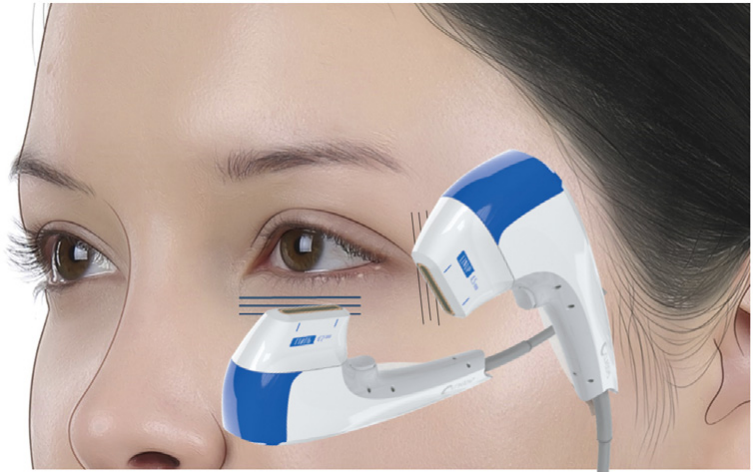
Each patient received 40 treatment shots on the upper eyelids (ULTRAFORMER MPT, Classys Inc., Korea), lower eyelids, and crow’s feet (20 shots per side), totaling 120 shots per patient.

Treatment was performed using the ULTRAFORMER MPT (Classys Inc., Seoul, South Korea) equipped with a 2.0-mm, 4-MHz transducer specifically designed for periocular application. The device was set at an energy level of 0.2 J (Fig. 1).

Each patient received a total of 120 shots, distributed as follows:

Upper eyelids: 40 shots (20 per side).

Lower eyelids and crow’s feet: 80 shots (40 per side).

The transducer was held perpendicular to the skin surface and moved continuously to avoid energy overlap or thermal stacking. Corneal shields were inserted in both eyes to protect the ocular surface during treatment. No additional cooling or analgesia was required.

### Outcome evaluation

#### Clinical assessment

Participants were evaluated at baseline (day of treatment) and 12 weeks post-treatment. Standardized digital photographs were obtained using identical camera settings, patient positioning, and lighting conditions, capturing both frontal and 45° angled views of the face.

Two board-certified aesthetic clinicians, blinded to treatment sequence, independently compared pre- and post-treatment photographs. They identified post-treatment images and graded overall improvement, eyelid tightening, and crow’s feet reduction using a four-point improvement scale: 1 = no or minimal improvement (<25 %), 2 = mild improvement (26–50 %), 3 = moderate improvement (51–75 %), 4 = marked improvement (>75 %).

Any adverse effects (e.g., erythema, edema, ecchymosis, pain) were recorded immediately post-treatment and at the 12-week follow-up visit.

#### Objective measurement

Objective eyelid tightening was quantified by measuring the average lid length (ALL)—the vertical distance between the eyebrow’s inferior margin and the upper eyelid rim—on high-resolution frontal photographs.

A perpendicular line was drawn from the midpoint between the medial and lateral canthus, and distances were measured using calibrated digital analysis software (ImageJ, National Institutes of Health, USA).

The mean ALL was calculated at baseline and 12 weeks post-treatment. Statistical analysis was performed using a paired t-test to determine significance (*p* < 0.05).

#### Patient-reported outcomes

Each participant completed a self-assessment questionnaire at 12 weeks post-treatment. Using the same four-point scale, patients rated perceived improvement in eyelid tightness and crow’s feet, and indicated overall satisfaction and willingness to undergo the treatment again.

Discomfort during treatment was rated on a 10-point Visual Analog Scale (VAS), where 0 = no pain and 10 = unbearable pain.

## Results

All 34 participants completed the study and were available for follow-up at 12 weeks. Their ages ranged from 31 to 67 years; 27 patients had Fitzpatrick skin type III and seven had type IV.

### Clinical assessment

**Figure 2:**
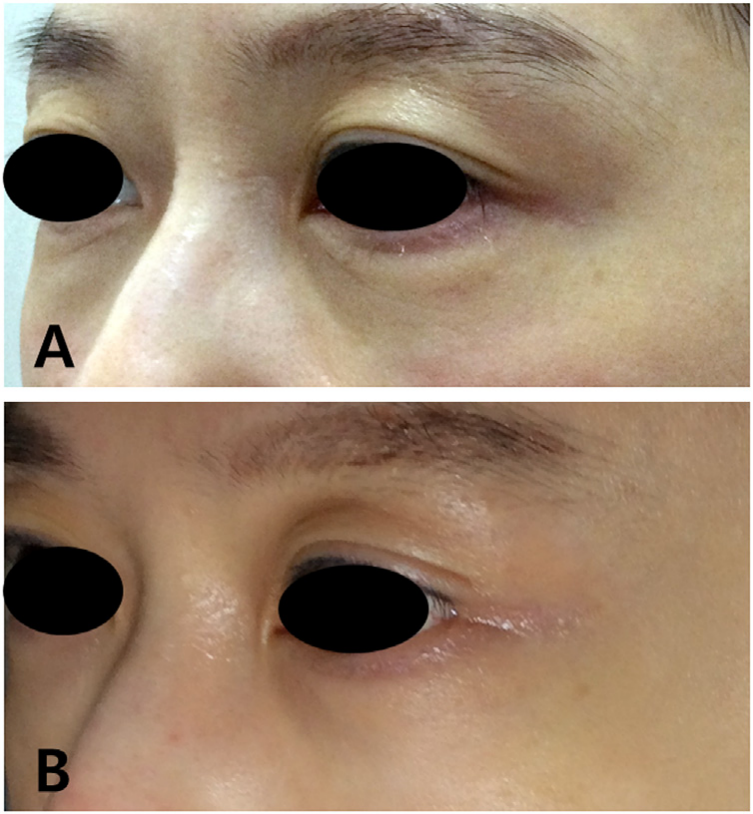
Lateral view of a patient before (A) and 12 weeks after treatment (B) demonstrating improvement in crow’s feet.

**Figure 3:**
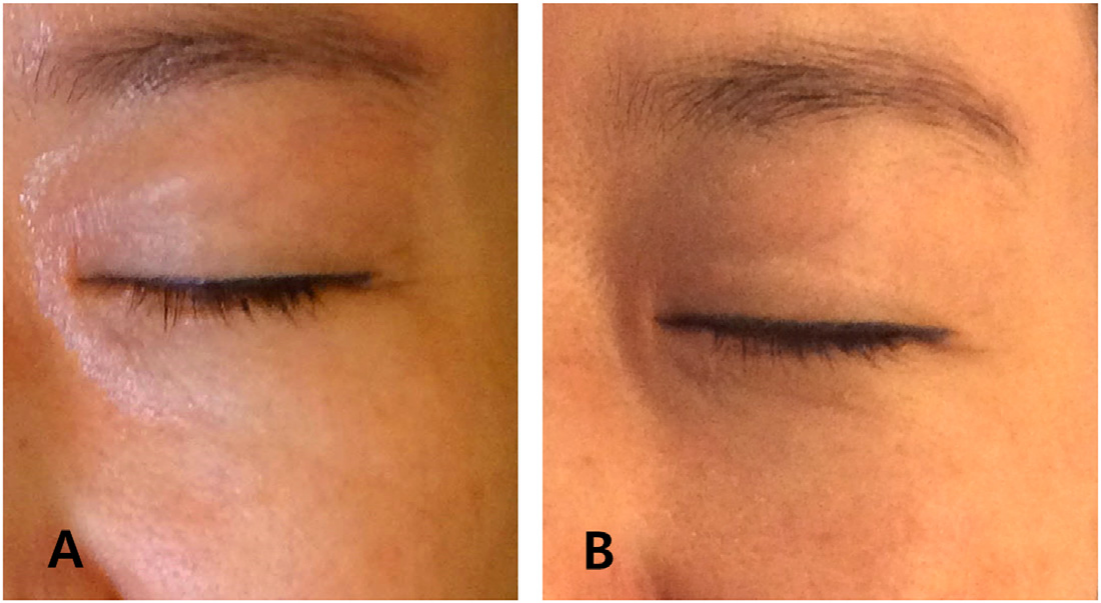
Frontal view of a patient before (A) and 12 weeks after treatment (B).

Clinician evaluation identified post-treatment images correctly in 26 of 34 cases (76 %) (Figure 2, Figure 3). The average clinician scores were:

Overall improvement: 2.10 ± 0.3.

Eyelid tightening: 2.30 ± 0.4.

Crow’s feet reduction: 2.40 ± 0.3.

In 10 cases (24 %), no notable difference was observed between pre- and post-treatment images, all of which were assigned a score of one.

### Patient-reported outcomes

Twenty-six participants (76 %) reported visible improvement in the periocular region 12 weeks after HIFU. The mean patient-rated scores were:

Overall improvement: 2.25 ± 0.3.

Eyelid tightening: 2.10 ± 0.4.

Crow’s feet reduction: 1.95 ± 0.3.

The mean VAS pain score was 3.75 ± 1.6, indicating mild procedural discomfort. A total of 76 % of patients expressed willingness to undergo repeat treatment.

### Objective measurement

The mean ALL significantly decreased from 16.3 ± 2.42 mm at baseline to 15.2 ± 2.35 mm at 12 weeks, reflecting a mean reduction of 0.94 ± 0.34 mm (t (33) = 16.21, *p* < 0.0001).

This reduction demonstrates measurable tightening of upper eyelid skin.

### Safety

No serious adverse events occurred. Mild erythema and transient swelling were observed in several participants and resolved spontaneously within 3–4 days without intervention. No pigmentary changes, burns, or ocular complications were reported.

## Discussion

Eyelid sagging is a common aesthetic concern that may progress to functional ptosis if untreated. Depending on the severity, surgical procedures such as Müller’s muscle-conjunctival resection, levator palpebrae shortening, or brow/frontalis suspension are can be performed.^10^^,^^12^ However, these operations are invasive and carry potential complications including scarring, infection, hematoma, asymmetry, and even visual disturbance. Consequently, there is increasing demand for noninvasive rejuvenation procedures such as fractional lasers, intense pulsed light, and radiofrequency systems that stimulate dermal remodeling and collagen synthesis.^13^^,^^14^

Among these modalities, high-intensity focused ultrasound (HIFU) has emerged as a unique technology capable of inducing collagenesis through thermal coagulation and mechanical tissue contraction without epidermal damage.^15^ Multiple clinical studies have verified its efficacy and safety in facial tightening.^16^ Nevertheless, data specific to the periocular region remain scarce.^7^^,^^17^ Although eyebrow lifting via HIFU has been reported,^18^ those studies targeted the forehead rather than the eyelid itself. To our knowledge, few investigations have directly assessed the safety and rejuvenating effect of HIFU applied to the delicate eyelid area.

### Clinical significance of findings

Our results demonstrate that HIFU effectively improves periocular laxity. Both clinician and patient assessments exceeded a mean score of two on a four-point scale (2.30 ± 0.4 and 2.25 ± 0.3, respectively), indicating mild-to-moderate aesthetic improvement. The reduction in mean average lid length (ALL) by 0.94 mm (*p* < 0.0001) objectively confirmed eyelid tightening. These results support HIFU as a quantifiable and safe noninvasive method for upper and lower eyelid rejuvenation.

The biological mechanisms underlying this effect involve localized thermal injury and collagen denaturation within the dermis and superficial muscular aponeurotic system (SMAS), leading to immediate fiber contraction and subsequent neocollagenesis. Historically, the thermal and mechanical actions of ultrasound have been exploited for tumor ablation,^19^ but the technique’s adaptation for dermatologic use was pioneered by White et al.,^20^ who demonstrated selective collagen contraction at specific depths without surface damage.^21^ The Ultraformer system used in this study employs a 2.0-mm probe optimized for narrow anatomical spaces, facilitating precise energy delivery to periocular tissues while minimizing risk to the ocular surface.

### Safety considerations

Proper procedural technique is essential for patient safety. In our series, all treatments were completed without major complications. Use of corneal shields, controlled probe angulation, and continuous motion prevented corneal injury. Temporary erythema and mild pain resolved spontaneously within 3–4 days. No pigmentary alteration or visual disturbance occurred. These findings confirm that HIFU, when performed by experienced clinicians using protective measures, can be safely applied to the periocular area.

### Comparative and adjunctive perspectives

In patients presenting with eyelid sagging secondary to prior filler misplacement or overcorrection, HIFU may represent a noninvasive adjunct or alternative to enzymatic degradation.

A recent scoping review on hyaluronidase highlighted the limited quality of evidence supporting its use for reversing filler-related complications in the periorbital region.^22^ Given the risk of allergic reaction and the lack of standardized dosing for non-hyaluronic acid fillers, HIFU could be a suitable therapeutic option for selected patients with mild-to-moderate skin laxity where filler removal is contraindicated or ineffective.

### Study limitations and future directions

This study has several limitations. First, the sample size was modest (*n* = 34), which limits statistical power and generalizability.

Second, all participants were Korean women with Fitzpatrick skin types III–IV, restricting extrapolation to other ethnic groups or skin types.

Third, the absence of a control or sham-treated group prevents definitive attribution of the observed effects solely to HIFU.

Fourth, subjective assessment scales were used; future studies should incorporate validated outcome measures such as the *Global Aesthetic Improvement Scale* (GAIS) and objective tools like 3D stereophotogrammetry to improve reliability.

Finally, the follow-up period was limited to 12 weeks. Long-term prospective studies (6–12 months) and histologic investigations are warranted to evaluate the persistence of collagen remodeling and the need for maintenance sessions.

### Conclusion of discussion

In summary, this study provides clinical and quantitative evidence that HIFU offers measurable tightening and rejuvenation of both upper and lower eyelids, with high patient satisfaction and minimal downtime. With appropriate safety protocols, HIFU may serve as an effective noninvasive alternative to surgical blepharoplasty and as a complementary modality in comprehensive periocular rejuvenation strategies.

## Conclusion

High-intensity focused ultrasound (HIFU) demonstrated measurable efficacy in improving upper and lower eyelid laxity, achieving both clinical and patient-reported improvement with no major complications. The observed reduction in eyelid length and overall tightening confirm its potential as a reliable noninvasive alternative to surgical blepharoplasty for patients with mild to moderate eyelid sagging.

The treatment is characterized by a favorable safety profile, minimal downtime, and high patient satisfaction, provided that appropriate ocular protection and procedural techniques are observed. As a brand-agnostic energy-based platform, HIFU offers flexibility in clinical application across different patient populations and device types.

Future research should include larger, multicenter, and randomized controlled trials with extended follow-up to evaluate long-term durability, histologic correlation, and optimal treatment parameters. Integration of HIFU into multimodal rejuvenation protocols may further enhance periocular outcomes and expand its role as a cornerstone in aesthetic medicine.

## Funding

None.

## Ethical approval

Not required.

## Acknowledgments

This study was conducted in compliance with the Declaration of Helsinki.

## Financial disclosure

There is no financial disclosure to report.

## Declarations of competing interest

The author acknowledge that he has considered the conflict of interest statement included in the “Author Guidelines.” The author hereby certify that, to the best of his knowledge, that no aspect of his current personal or professional situation might reasonably be expected to significantly affect his views on the subject he is presenting.

## Data availability statements

The data is available by requesting to corresponding author.

## Author contributions

All authors have reviewed and approved the article for submission.

Conceptualization, **Yousun Hwang:** Writing—Original Draft Preparation. **Yousun Hwang, Jin-Hyun Kim; Massimo Vitale, Isaac Wong Kai Jie; Arash Jalali; Han Earl Lee, Eun-Jae Kim:** Writing—Review & Editing. **Kyu-Ho Yi, Isabella Rosellini, Massimo Vitale, Visualization, Yousun Hwang, Kyu-Ho Yi, Jesper Thulesen:** Supervision. **Kyu-Ho Yi, Yousun Hwang**.
